# Supplementary figures and images for: Evolutionary analyses of intrinsically disordered regions reveal widespread signals of conservation
Source: PLoS Comput Biol. 2024 Apr 25;20(4):e1012028. doi: 10.1371/journal.pcbi.1012028 (PMC11075841; doi:10.1371/journal.pcbi.1012028)

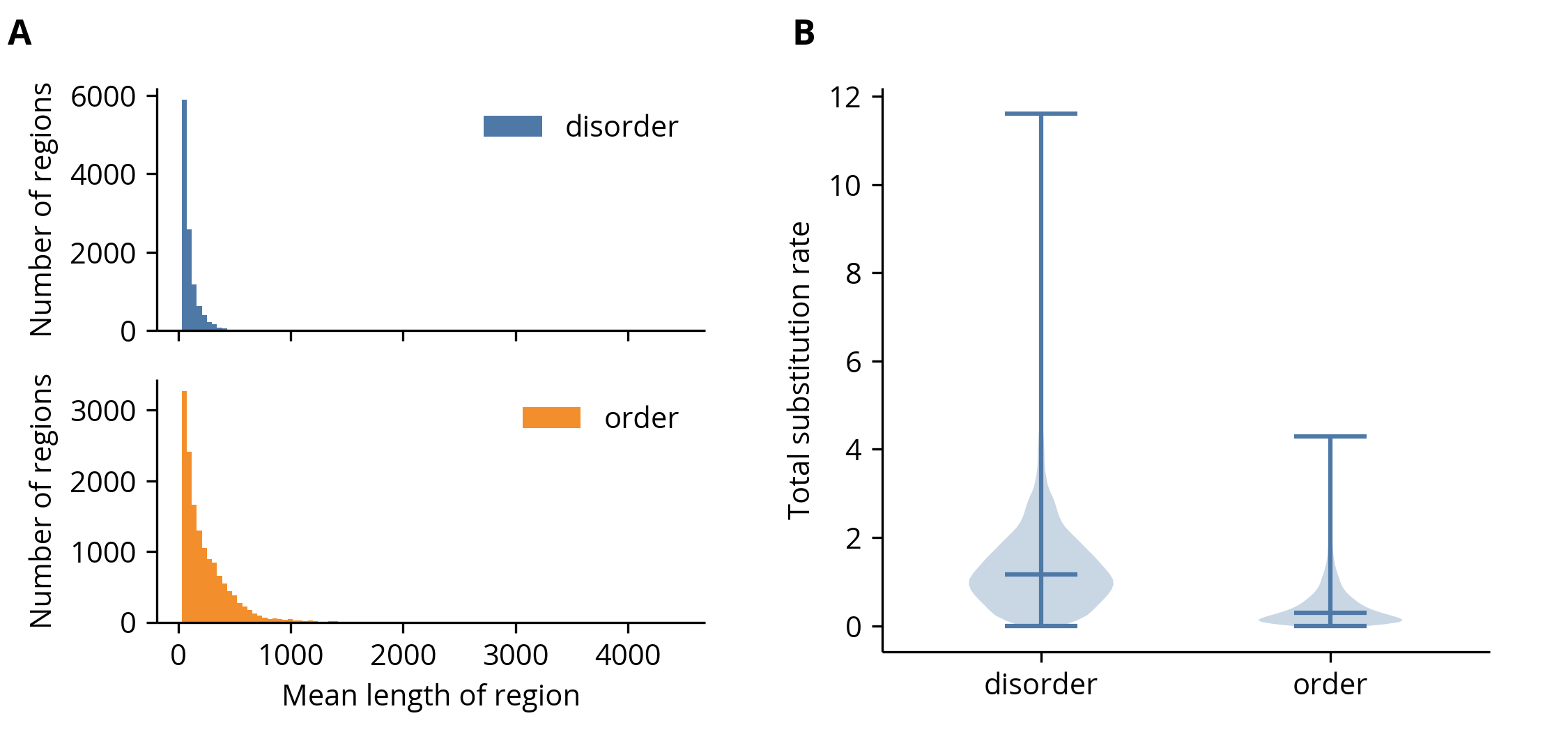

Supplement: S1 Fig — (A) Distribution of mean lengths of regions. (B) Violin plot of the sums of the average amino acid and indel substitution rates in the disorder and order regions. The substitution rates are significantly greater in the disorder regions than in the order regions (p < 1 × 10^−10^, Mann-Whitney U test). (TIFF) [file pcbi.1012028.s001.tiff]

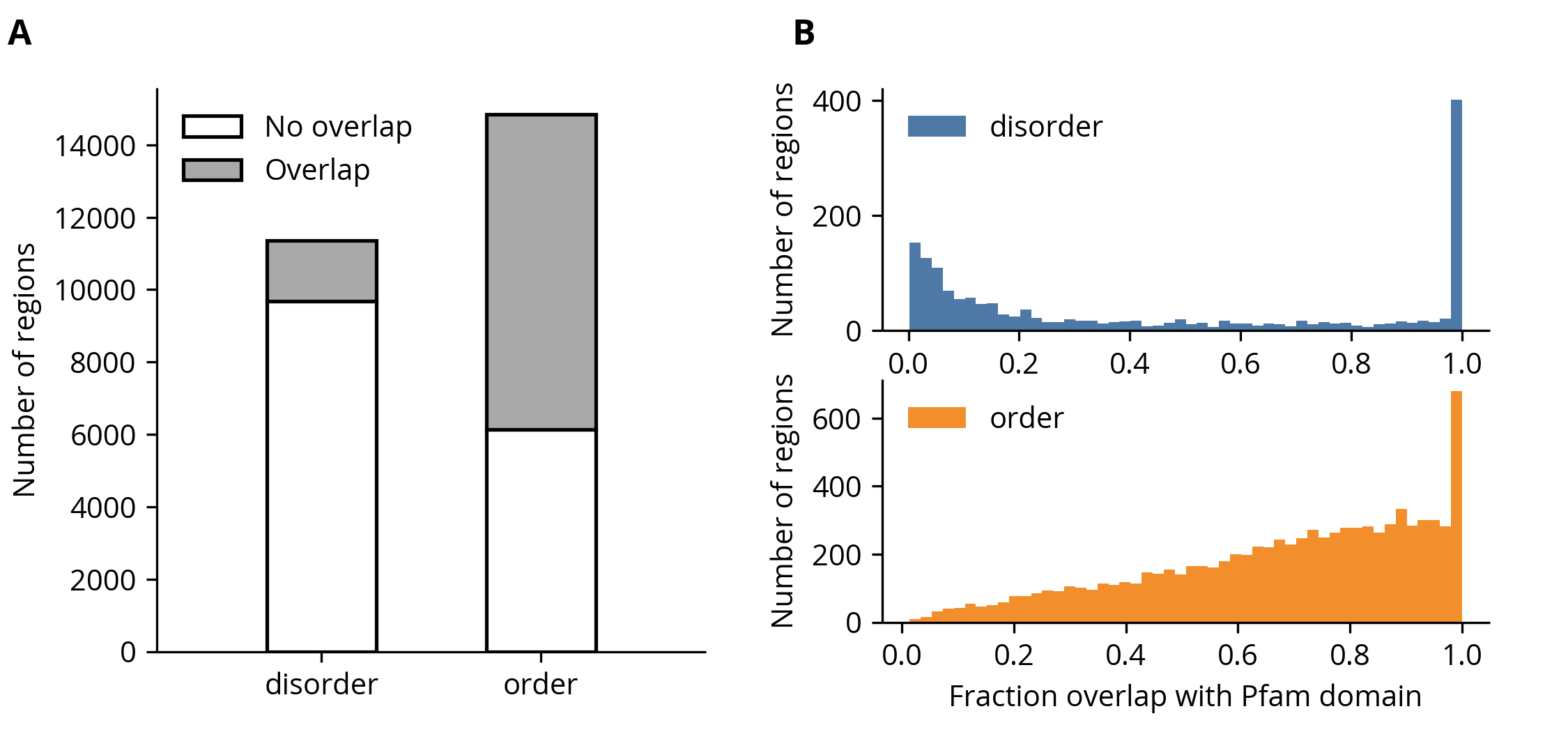

Supplement: S2 Fig — (A) Number of disorder and order regions with zero and non-zero overlap with any Pfam domain. The proportion of regions with no overlap is significantly greater for the disorder regions (p < 1 × 10^−10^, chi-squared test). (B) Histogram of the disorder and order regions’ overlaps with any Pfam domain. Regions with zero overlap are excluded to more clearly show the distribution. (TIFF) [file pcbi.1012028.s002.tiff]

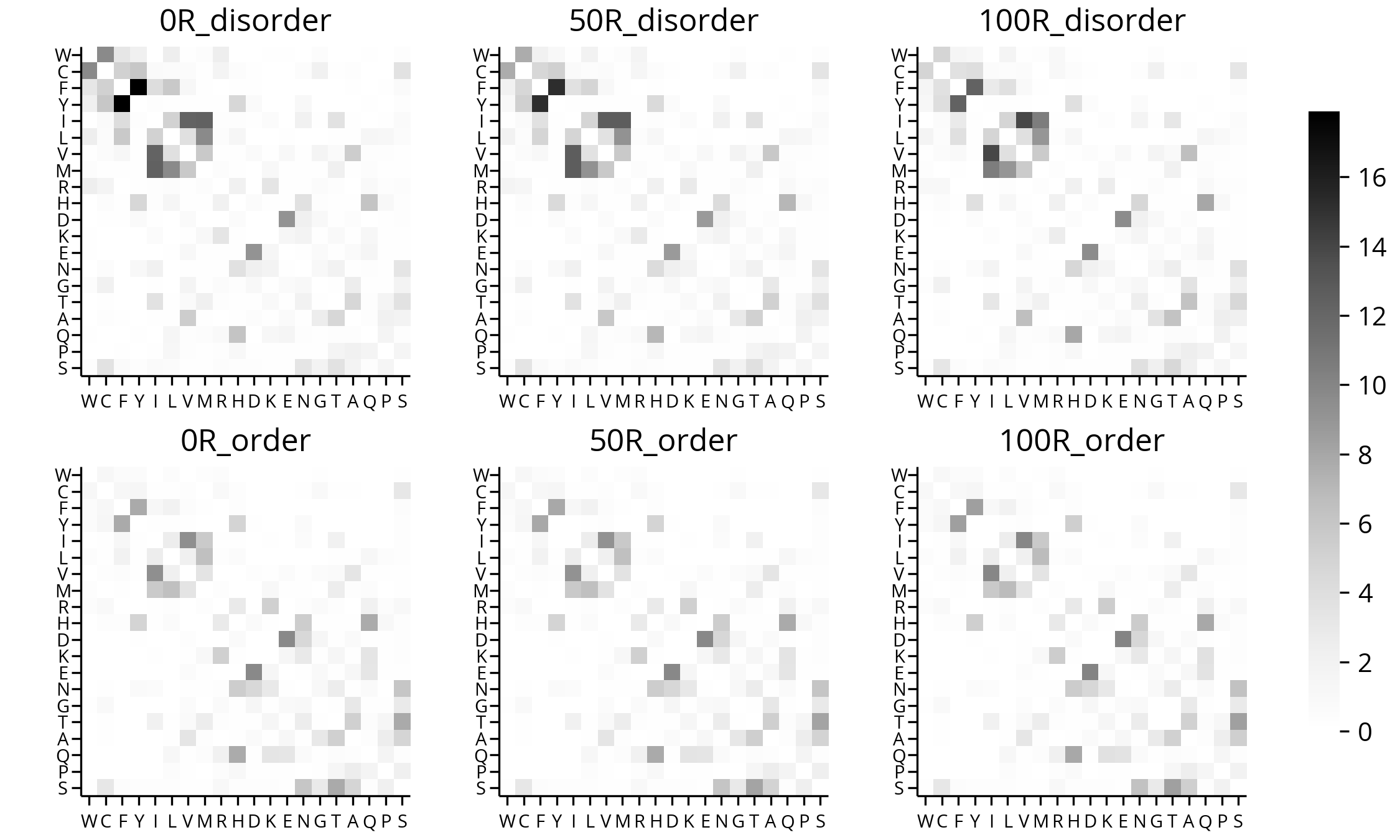

Supplement: S3 Fig — Each panel is a mean of the exchangeability coefficients fit to the meta-alignments yielded by a single sampling strategy (n = 25). The prefix and suffix in the title of each panel indicate the maximum gap fraction and region type of the columns in the meta-alignments, respectively. For example, the columns in the “50R_disorder” set of meta-alignments were fewer than 50% gaps and sampled from the disorder regions. (TIFF) [file pcbi.1012028.s003.tiff]

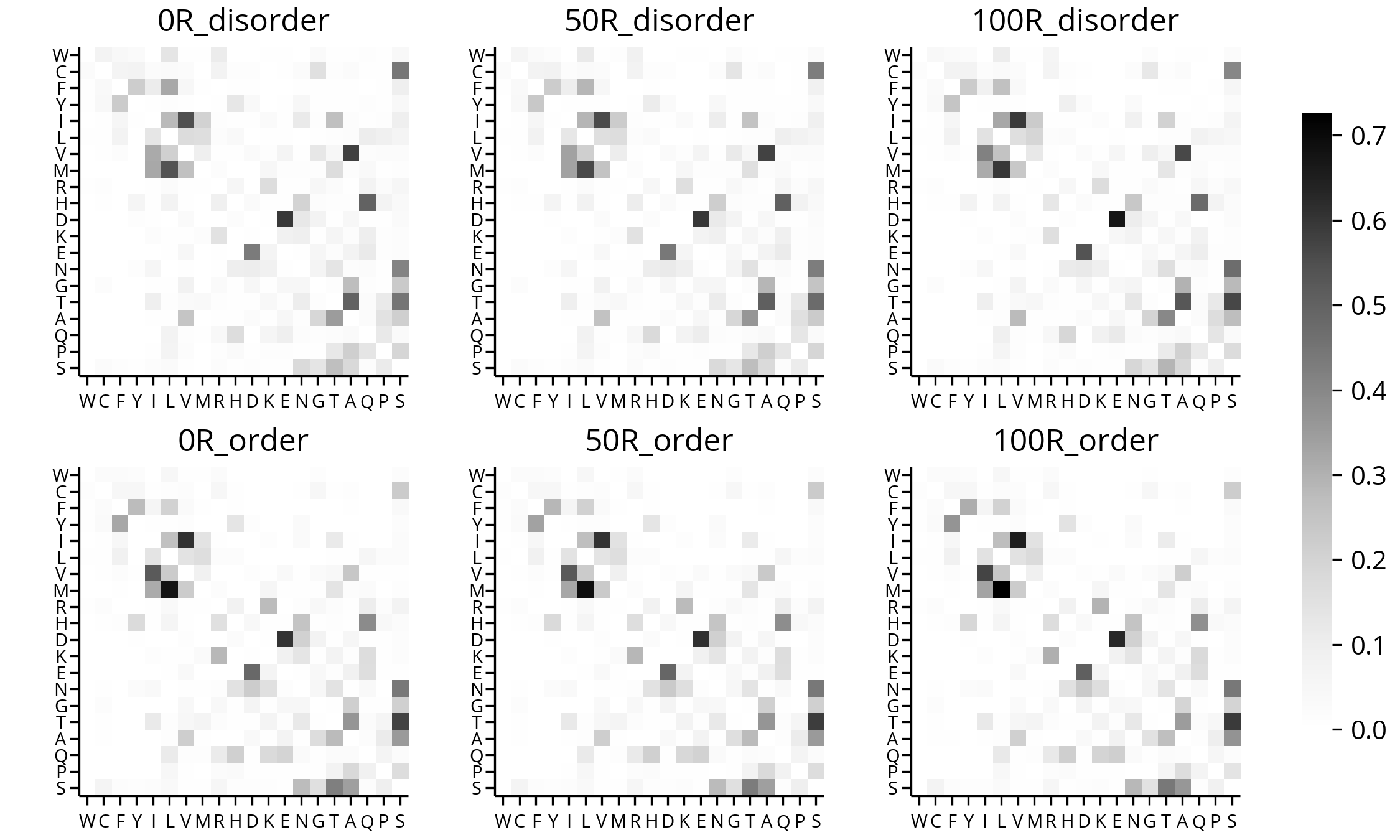

Supplement: S4 Fig — Each panel is a mean of the rate coefficients fit to the meta-alignments yielded by a single sampling strategy (n = 25). See S3 Fig for an explanation of the panel labels. (TIFF) [file pcbi.1012028.s004.tiff]

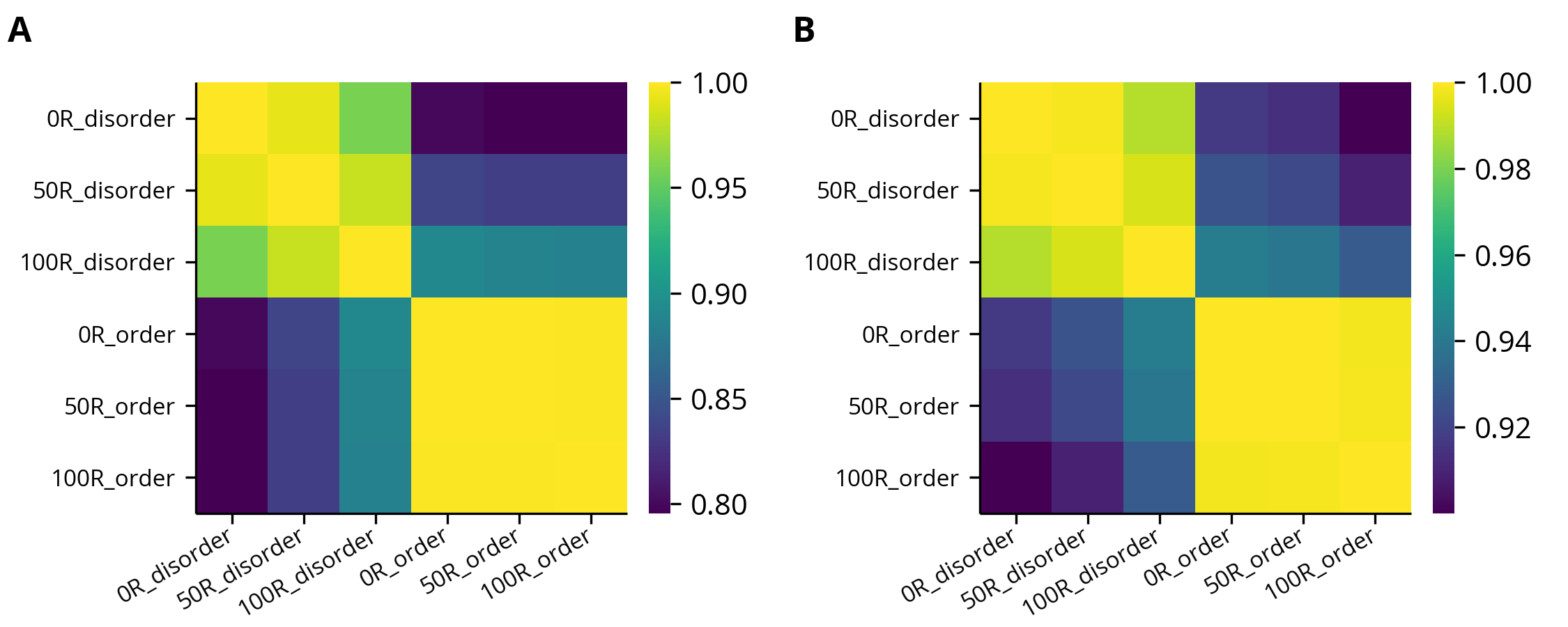

Supplement: S5 Fig — (A) Correlations between the mean exchangeability matrices in S3 Fig. (B) Correlations between the mean rate matrices in S4 Fig. (TIFF) [file pcbi.1012028.s005.tiff]

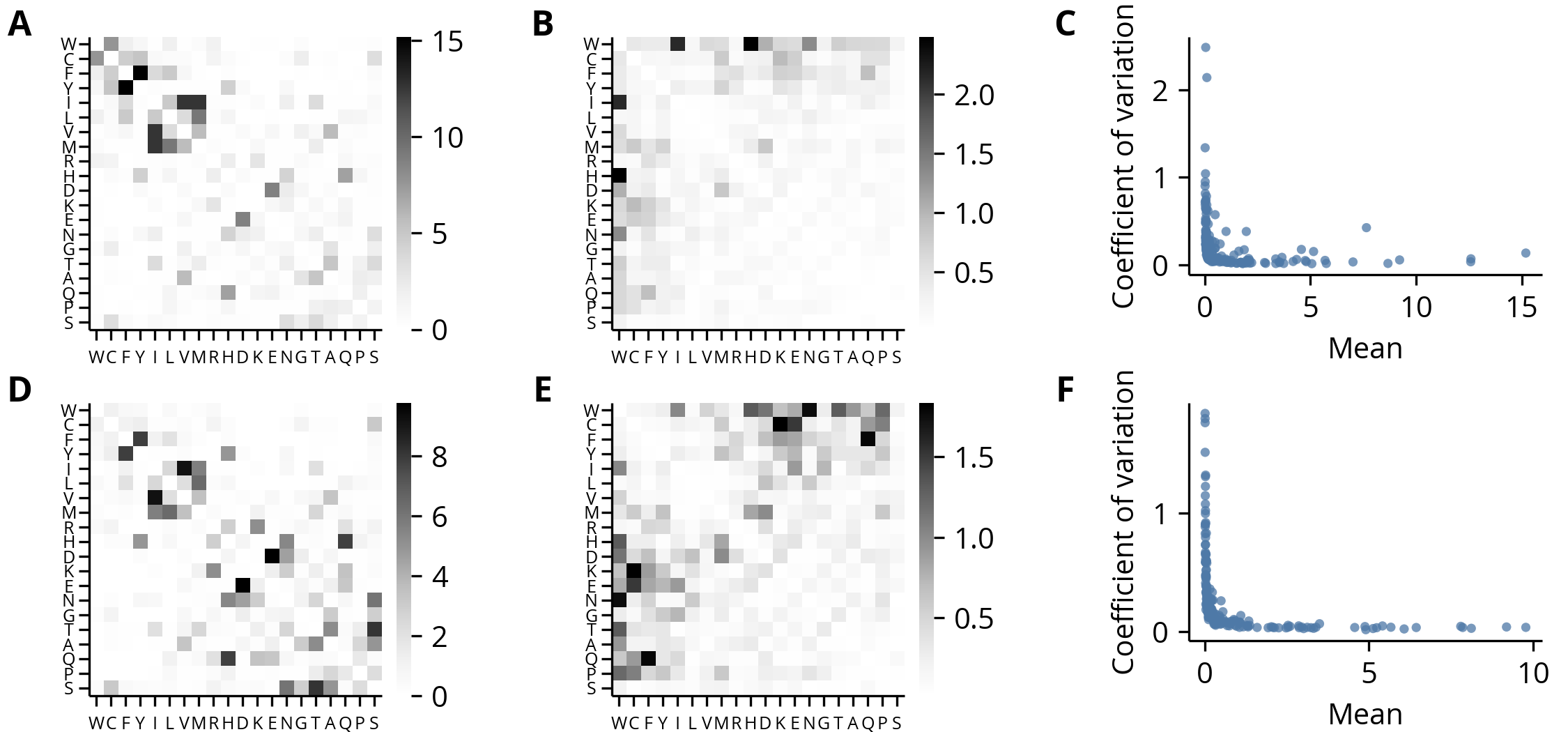

Supplement: S6 Fig — For all panels, the top and bottoms rows correspond to the 50R_disorder and 50R_order meta-alignment sets, respectively. (A, D) Mean exchangeability matrices. (B, E) Coefficients of variation (ratio of the standard deviation to the mean) of exchangeability matrices. (C, F) The coefficient of variation is inversely proportional to the mean, indicating the variation in the parameter estimates is constant relative to their magnitude. (TIFF) [file pcbi.1012028.s006.tiff]

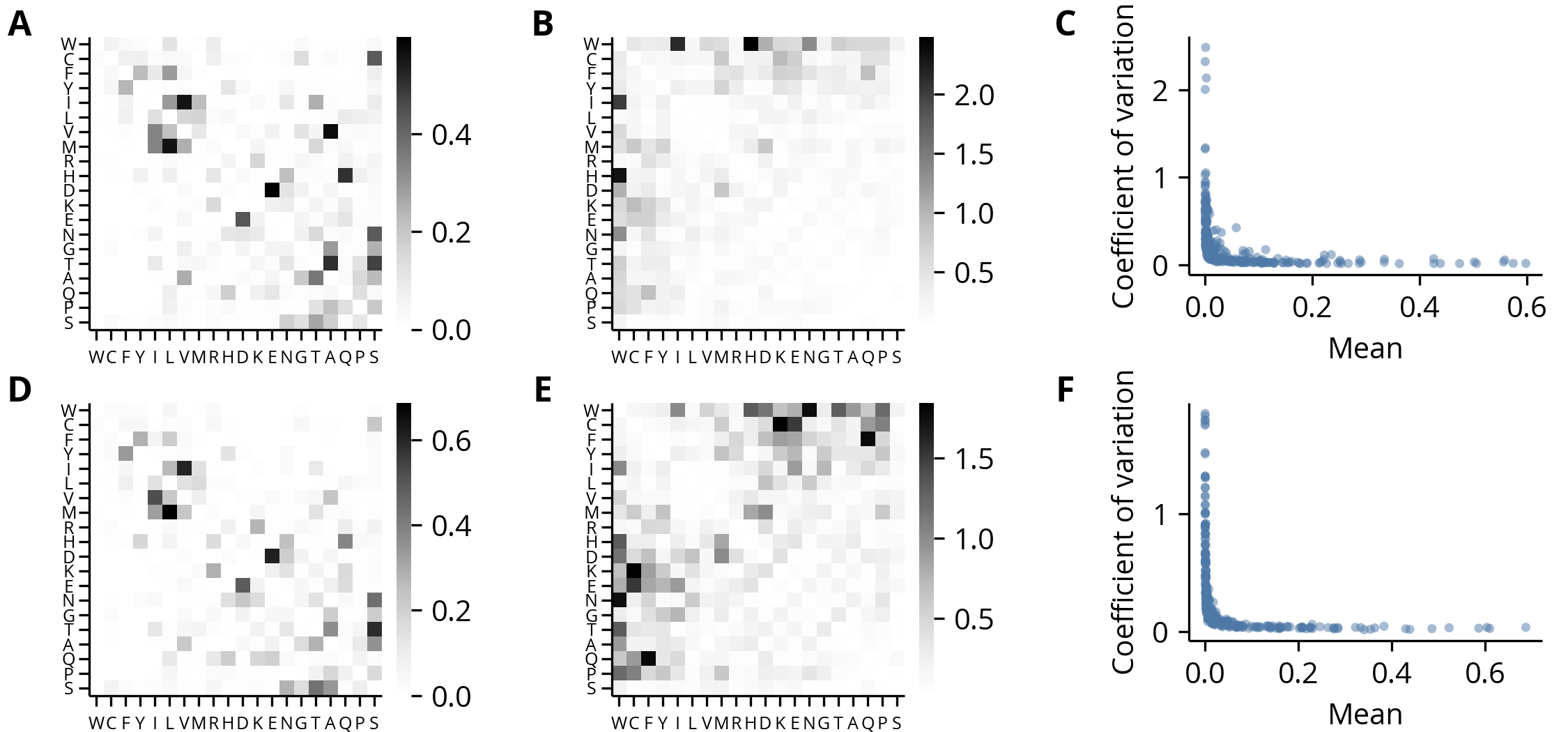

Supplement: S7 Fig — For all panels, the top and bottoms rows correspond to the 50R_disorder and 50R_order meta-alignment sets, respectively. (A, D) Mean rate matrices. (B, E) Coefficients of variation (ratio of the standard deviation to the mean) of rate matrices. (C, F) The coefficient of variation is inversely proportional to the mean, indicating the variation in the parameter estimates is constant relative to their magnitude. (TIFF) [file pcbi.1012028.s007.tiff]

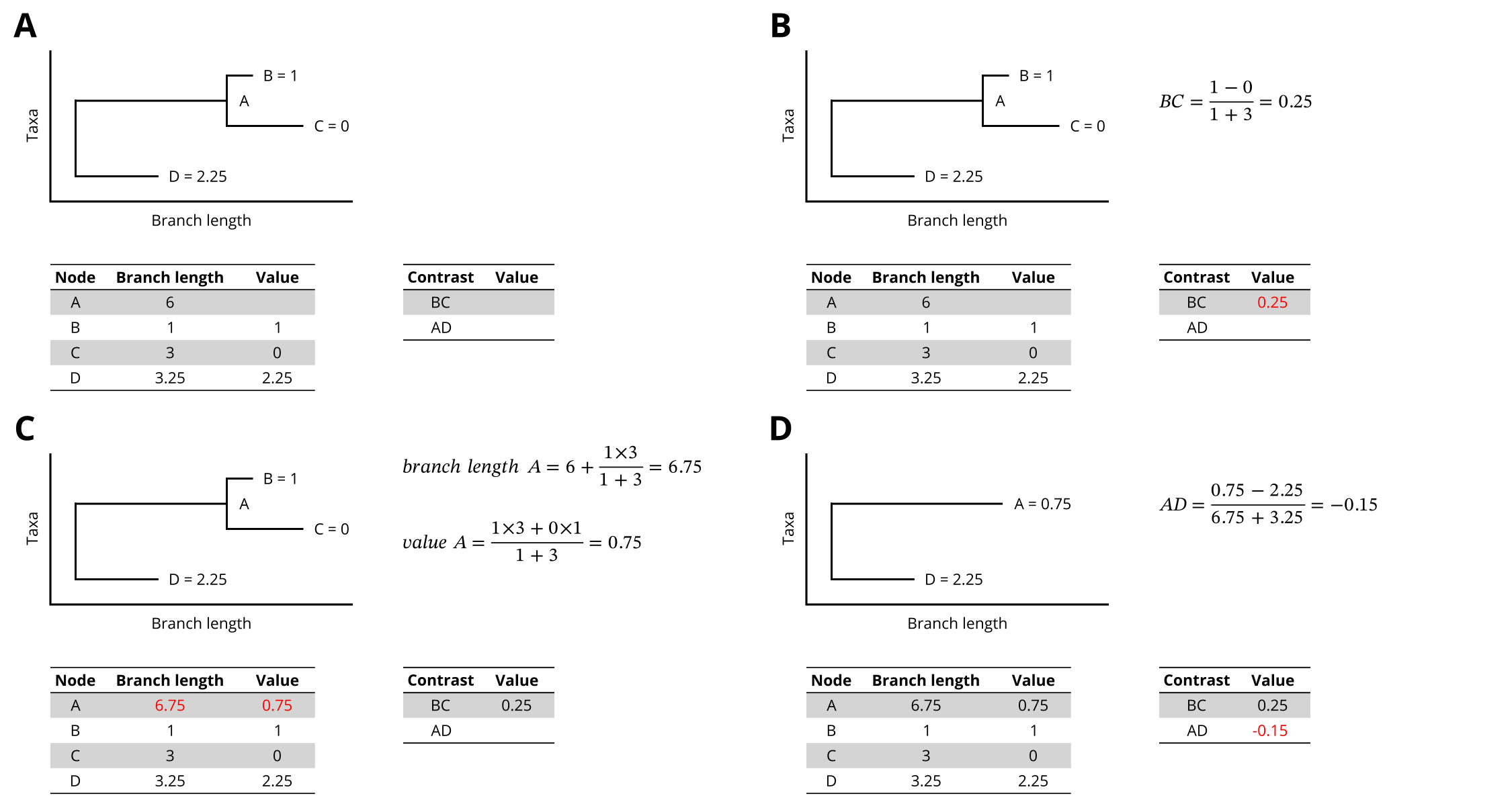

Supplement: S8 Fig — (A) Initial state of tree with three tips. Values of traits at each tip are indicated on the tree and in the table. (B) Calculation of first contrast between tips B and C. (C) Inference of trait value at internal node A. Its branch length is increased to account for the uncertainty in the estimation of its value. (D) Calculation of second contrast between tip D and internal node A. (TIFF) [file pcbi.1012028.s008.tiff]

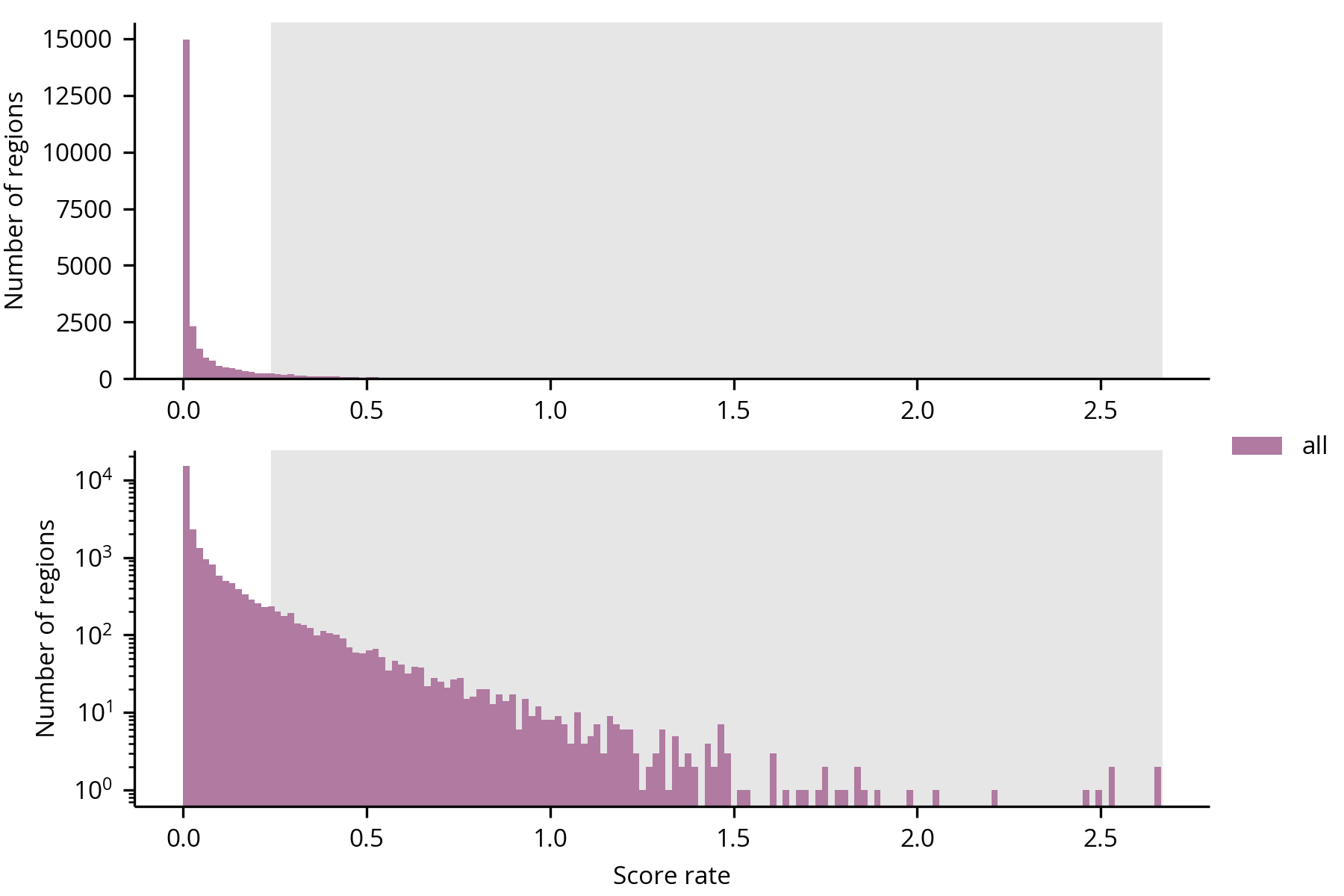

Supplement: S9 Fig — The grey interval indicates the upper decile of the distribution across both disorder and order regions, which was used as the input set for the GO term enrichment analysis. (TIFF) [file pcbi.1012028.s009.tiff]

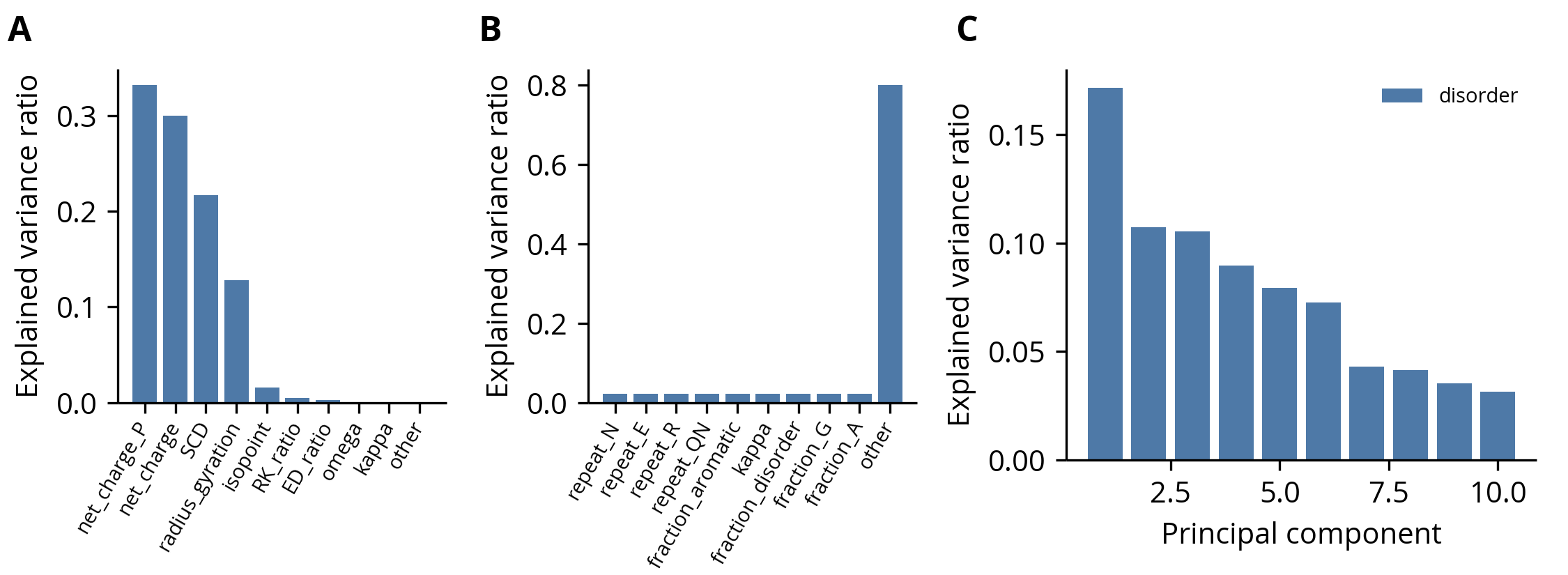

Supplement: S10 Fig — (A) Variance ratios before normalization. (B) Variance ratios after normalization. (C) Scree plot of the explained variance ratio by PC. (TIFF) [file pcbi.1012028.s010.tiff]

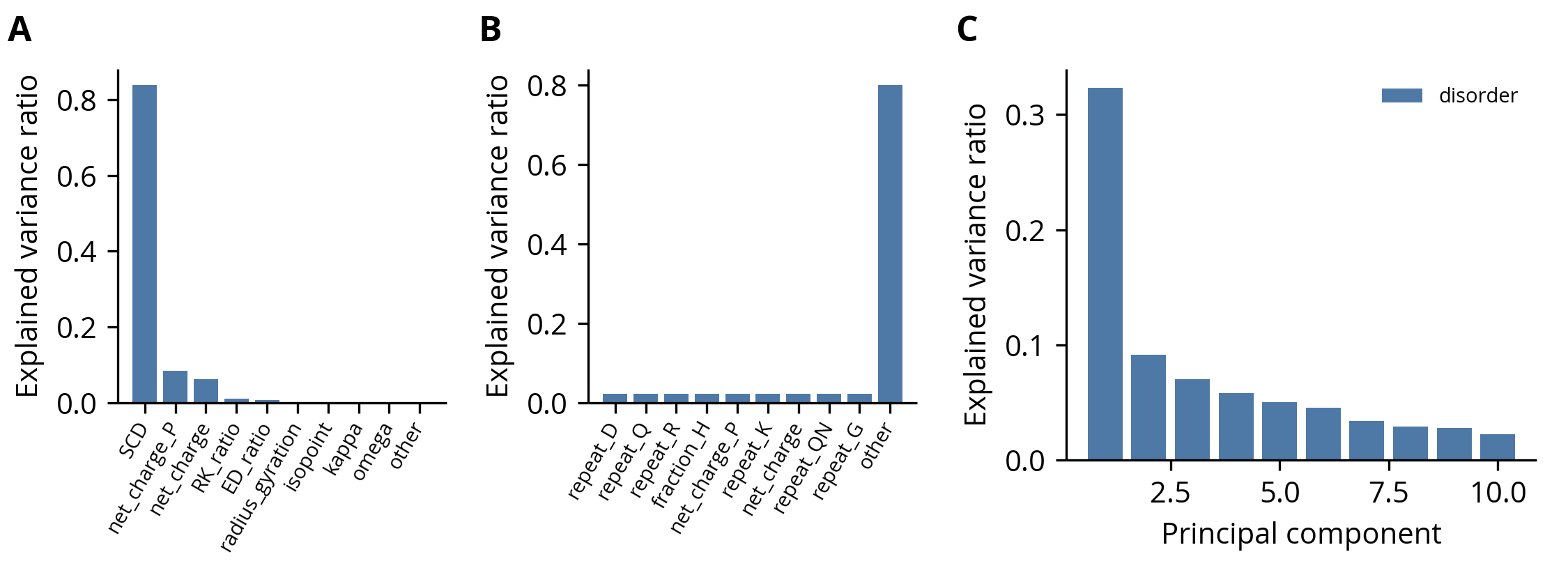

Supplement: S11 Fig — (A) Variance ratios before normalization. (B) Variance ratios after normalization. (C) Scree plot of the explained variance ratio by PC. (TIFF) [file pcbi.1012028.s011.tiff]

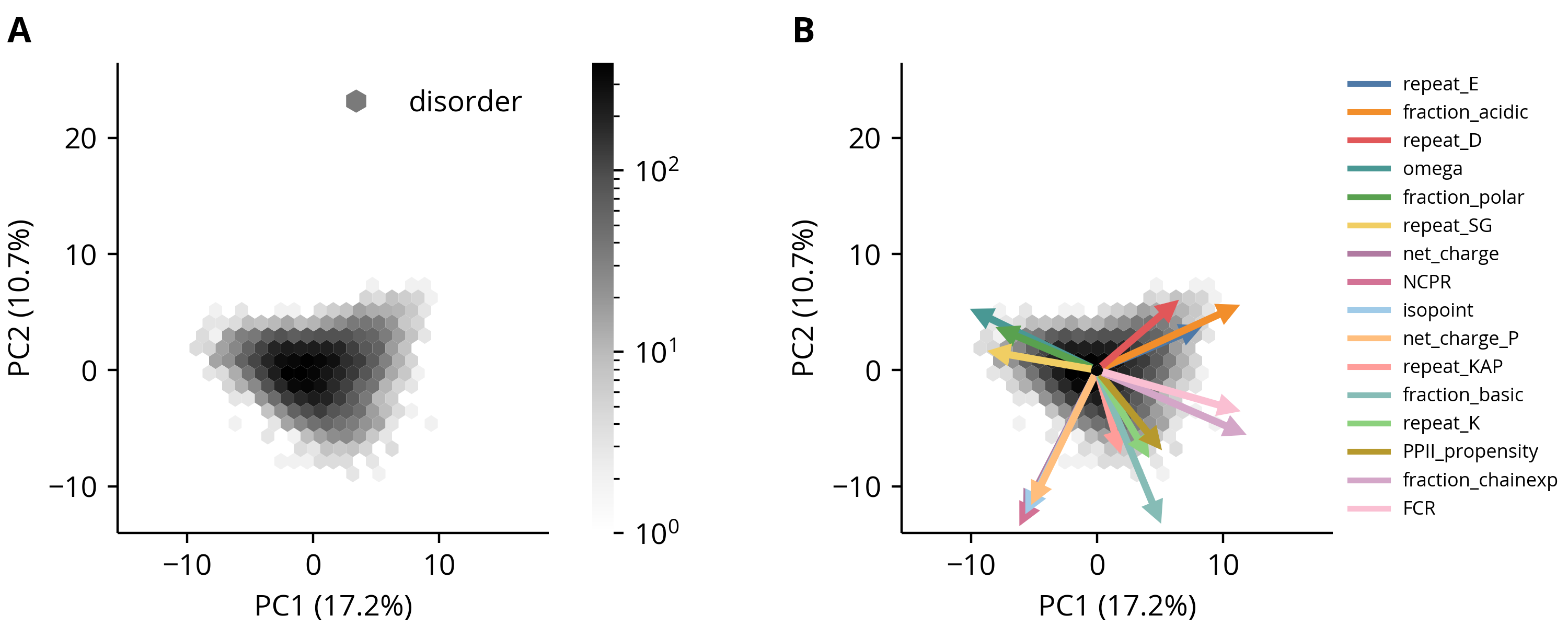

Supplement: S12 Fig — (A) The first two PCs of the disorder regions’ feature root distributions. The explained variance percentage of each component is indicated in parentheses in the axis labels. (B) The same plot as panel A with the projections of original variables onto the components shown as arrows. Only the 16 features with the largest projections are shown. Scaling of the arrows is arbitrary. (TIFF) [file pcbi.1012028.s012.tiff]

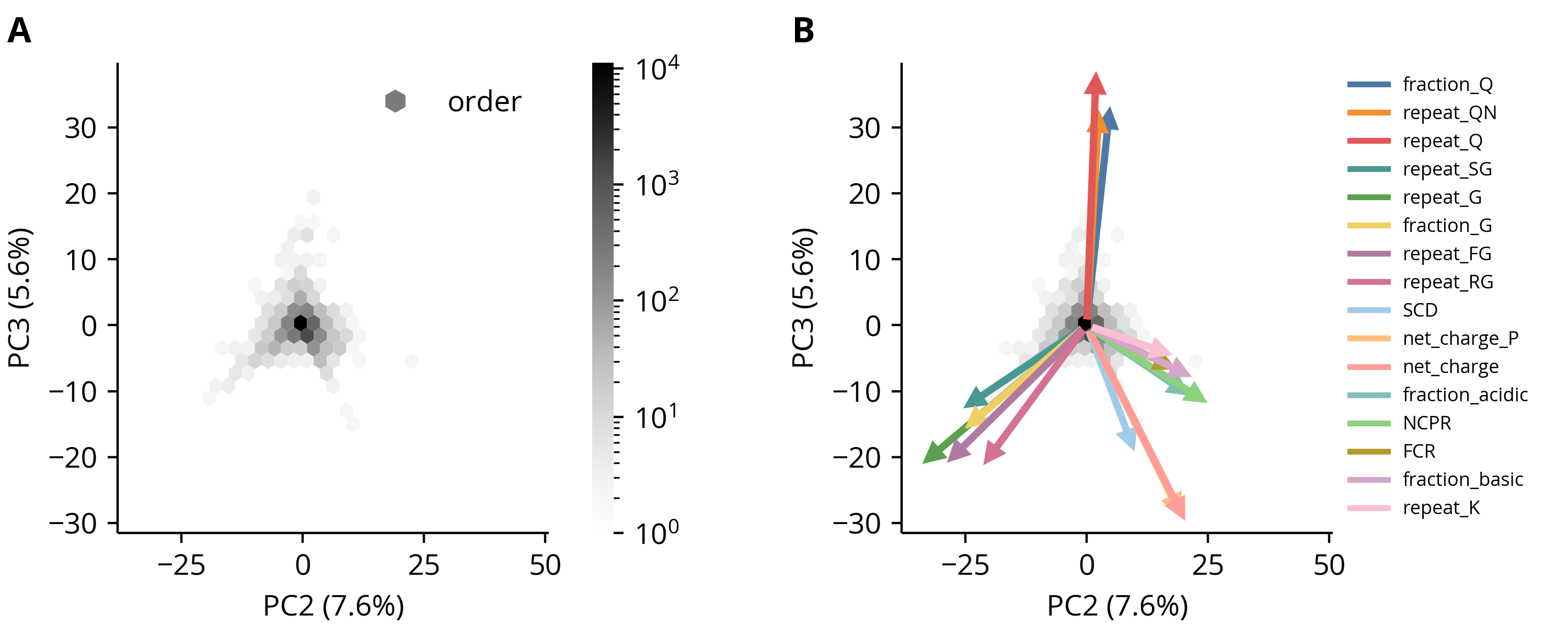

Supplement: S13 Fig — (A) The second and third PCs of the order regions’ feature rate distributions. The explained variance percentage of each component is indicated in parentheses in the axis labels. (B) The same plot as panel B with the projections of original variables onto the components shown as arrows. Only the 16 features with the largest projections are shown. Scaling of the arrows is arbitrary. (TIFF) [file pcbi.1012028.s013.tiff]

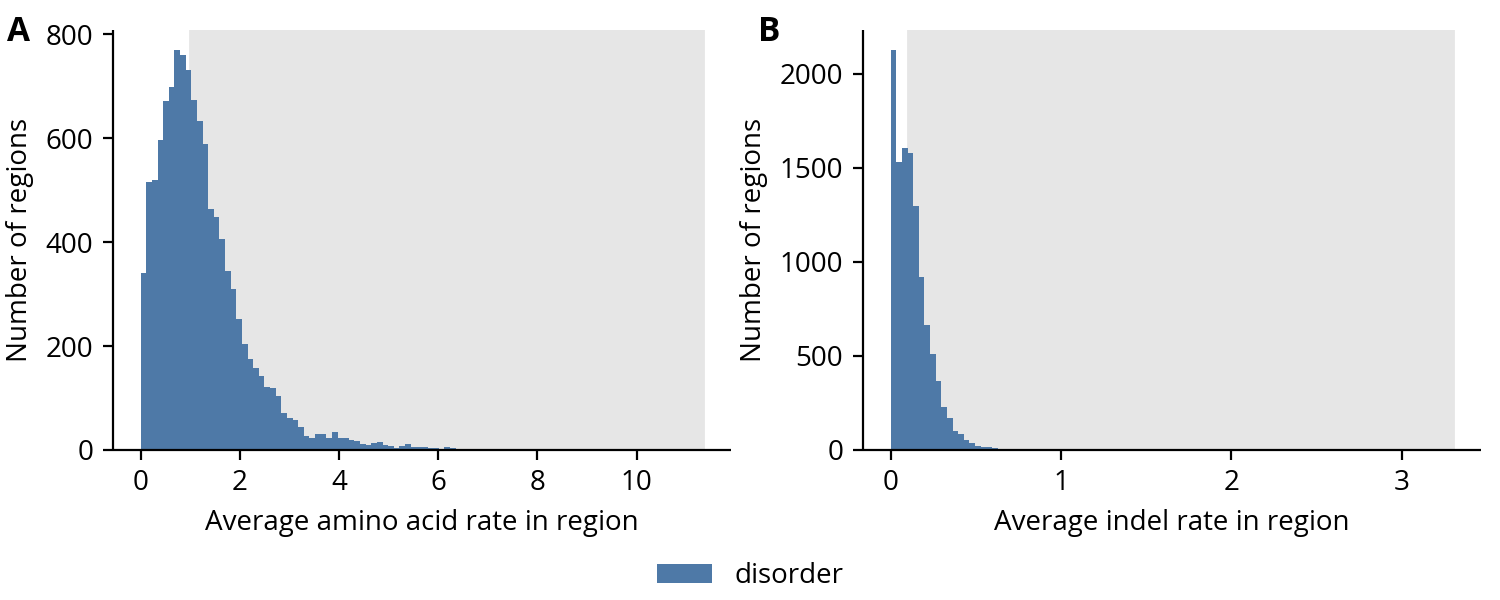

Supplement: S14 Fig — (A) Average amino acid rates in regions. (B) Average indel rates in regions. For both panels, the grey intervals correspond to the subsets of rapidly evolving regions used for the clustering and GO term enrichment analyses. 5892 (52%) and 6052 (53%) regions pass the amino acid and indel rate cutoffs, respectively, and 7607 (67%) of regions pass either. (TIFF) [file pcbi.1012028.s014.tiff]

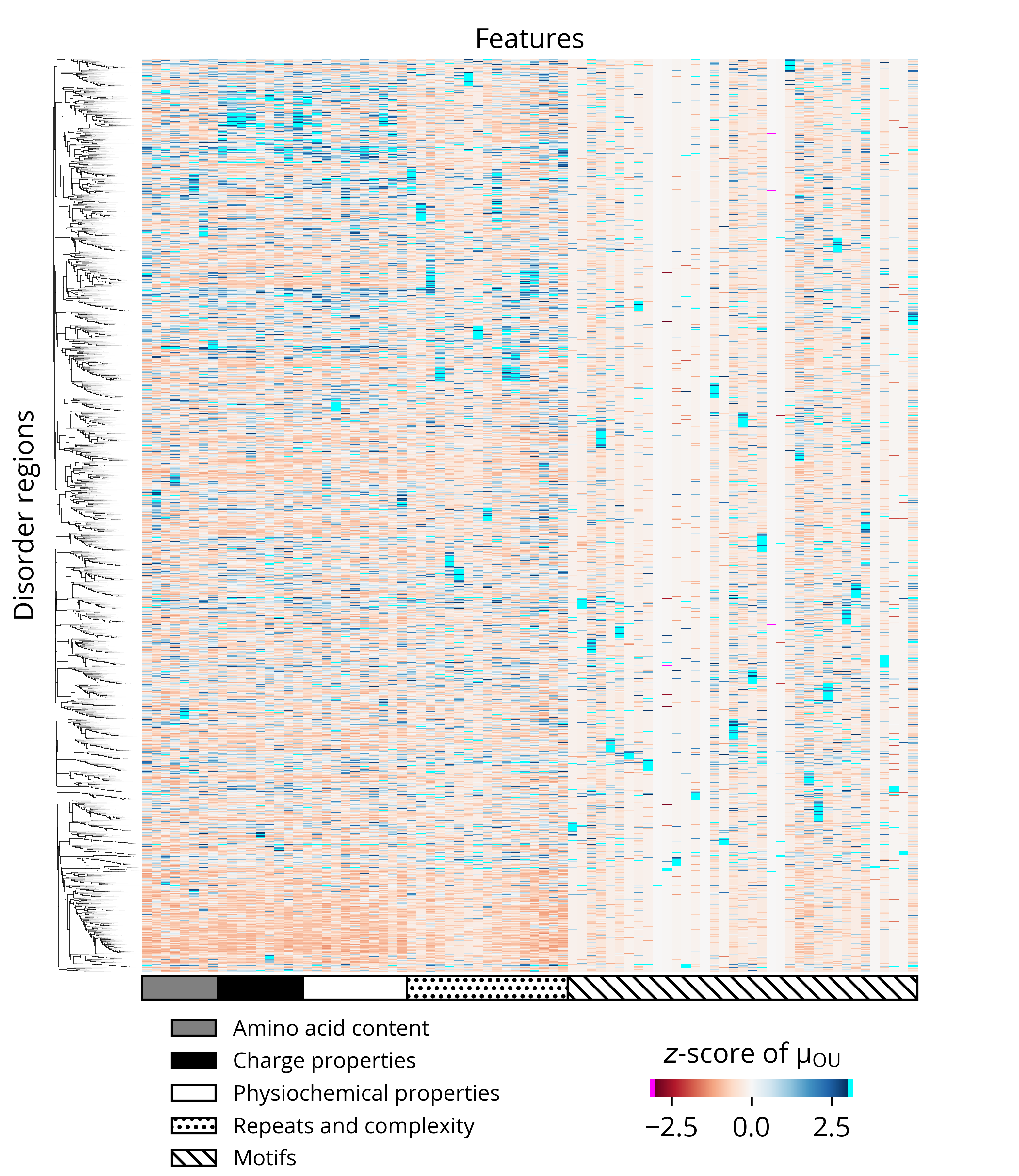

Supplement: S15 Fig — The optimal values of the OU model (μOU) are clustered with the same method as the signatures derived from the log likelihood ratios in Fig 7. The optimal values are expressed as z-scores relative to each feature distribution in the subset of rapidly evolving disorder regions. Values below or above negative or positive three are indicated with magenta and cyan, respectively. (TIFF) [file pcbi.1012028.s015.tiff]
