# Supplementary material for: Evolutionary analyses of intrinsically disordered regions reveal widespread signals of conservation
Source: PLoS Comput Biol. 2024 Apr 25;20(4):e1012028. doi: 10.1371/journal.pcbi.1012028 (PMC11075841; doi:10.1371/journal.pcbi.1012028)
Supplement: Attachment — Submitted filename: review_response.pdf [file pcbi.1012028.s019.pdf]

March 6, 2024

Dear Drs. Csikász-Nagy and Zhang,

Thank you for the opportunity to submit a revised manuscript of "Evolutionary analyses of intrinsically disordered regions reveal widespread signals of conservation" for publication in *PLOS Computational Biology*. We appreciate the time and effort that the Reviewers have dedicated to their thorough reading of our manuscript and are grateful for their feedback for improving the impact and presentation of our work. We have incorporated many of the suggestions made by the Reviewers, which are highlighted in the marked-up version of the manuscript. Below we address each of the Reviewers' comments in detail.

COMMENTS FROM REVIEWER 1

Comment 1:

However, some of the stated conclusions in the text are strong given the underlying evidence and caveats of the study. The limitations of the analyses are quite thoroughly documented in the discussion, but more explicit referral to this section and less overstatement of the evidence (in the main text/pre-discussion) would be helpful to understand the biology and remaining open questions. E.g. there should be a more explicit referral to what I see as the two major caveats of the study: a) that there are artefacts associated with applying continuous models of trait evolution to discrete data, and b) that there is a dependence of the method on substitution matrices inferred from sequence alignments.

Response to Comment 1:

Thank you for this comment. Though we do address these issues in the Discussion, we agree it would be helpful to reference these sections more directly in the preceding text. We have added a sentence at the end of the Introduction mentioning a) the limitations of the continuous models of trait evolution and another sentence in the Results mentioning b) the dependence of our inferred substitution matrices on those used to align the sequences in the first place.

Comment 2:

Fig. 2/intrinsic disorder poorly conserved in some proteins:

- there's no explicit mention of the error associated with disorder predictions and how this can impact the variable disorder scores across phylogenies. the authors should rule out the possibility of a technical artefact before claiming that the proteins that contain these IDRs have rapidly evolving structural states. this may especially be the case since the disorder scores can be variable despite high levels of sequence identity (line 172-173).
- though some examples are given in fig. 2, it would be helpful to know, e.g. by the authors' criteria, what proportion of the analyzed IDRs have poorly conserved disorder, and how this compares to previous studies in other species?

Response to Comment 2:

Thank you for raising these concerns. As our method for assessing the structural state of residues is based on a predictive model, it is possible the observed variability is a technical

artifact rather than a reflection of true differences in the sequences' propensity for disorder. However, a recent assessment of disorder predictors has shown the best available predictors can discriminate between disordered and structured residues with a fairly high accuracy (Necci *et al.*: 10.1038/s41592-021-01117-3). For example, as indicated in Fig. 3g of the referenced work, our chosen predictor, AUCpreD, has an ROC-AUC of 0.906. For comparison, perfect and random predictors have values of 1 and 0.5, respectively. More concretely, Supplementary Table 9 shows that at an MCC-optimal threshold, AUCpreD has true and false positive rates of 0.646 and 0.122, respectively, which translates to an 84% posterior probability that a predicted label of disorder is accurate. Given these values, it is unlikely the observed variability is solely artifactual. We have, however, added a sentence to the referenced paragraph that briefly summarizes the previous discussion.

Regarding the fraction of IDRs with poorly conserved disorder, we have chosen to not calculate this value, as we believe setting an arbitrary threshold would not strengthen our argument in this section. Our intention was instead to highlight our observation that some regions with relatively conserved sequences have highly variable disorder scores, illustrating that disorder itself can also be a rapidly evolving property. Additionally, the histogram of disorder score rates in Fig S8 shows a distribution with a long tail and no clean breaks between regions with conserved and rapidly evolving disorder. We are also unaware of any other studies that have performed a similar analysis.

Comment 3:
Fig. 5/Signals of feature conservation widespread in IDRs
- it would be helpful to know the number of IDRs (/total percentage) that pass the threshold for minimum amount of divergence? Perhaps just an inset in the grey box in Fig S13 or in fig. legend

Response to Comment 3:
We have added these values in the legend for Fig S13. We have also added the total percentage passing either cutoff in the corresponding section in the Methods as well as corrected a typo in the total number.

Comment 4:
Fig. 6: although a positive and negative example are presented, the second example in particular does not really serve as validation, since we do not in fact know if the low log likelihood ratios of glutamines in this region are suggestive of unconstrained glutamine repeat expansion/contraction. The fact that it is an unannotated gene does not count as evidence for this. It would be more convincing if the authors could present a validated example, perhaps from a previously studied drosophila IDR.

Response to Comment 4:
Thank you for this suggestion. We do not intend for the examples in Fig 6 to serve as validation *per se* but rather to illustrate the ability of our method to reveal statistically significant signals of conservation which are not readily apparent by eye. For example, though both alignments contain extensive polyglutamine expansions/contractions, only one is consistent with a model of conservation. Additionally, in the following discussion, we

are careful to emphasize the value of this method as a hypothesis-generating procedure when used with appropriate context.

Comment 5:
Fig. 7/clustering analysis: there could be several reasons other than a difference between multi-cellular and uni-cellular IDR evolution for the lack of associated function in clusters:
- given the GO terms in table 1, there seem to be some fairly general terms that are enriched such as "molecular function activator activity" -- if the authors limited the GO analysis to terms that do not apply to most drosophila genes, would they find more specific enrichment?
- the log ratio scores do not contain information about the directionality of the molecular feature conservation (e.g. is it the depletion that's conserved, or the enrichment of that feature?) -- could this decrease the resolution/interpretability of the analysis?
- how does the diversity of included species affect the signal of the assay? is there "enough" divergence? how does it compare to the previous study in yeast? can the authors provide more information on how the drosophila species were chosen in the methods (aside from the reference)?
- how does a clustering of physicochemical features (with no evolutionary information) compare to the results from the OU/BM analysis? Is there any more information gleaned from the phylogenetic modeling (e.g. more enrichment for GO terms in the clusters)?

Response to Comment 5:
Thank you for raising these excellent questions. We respond to each in turn below.

Though filtering can increase the power of FDR control procedures, we doubt it would substantially change our conclusions in this case. For example, in our term enrichment analysis, we conducted 25,807 tests using regions as the sampling unit. Of these, only 39 were for terms associated with over 5,000 regions. (All were for “biological process,” so each cluster was tested for enrichment in this term.) For comparison, Zarin *et al.* filtered their terms to those that applied to a maximum of 5,000 genes. Because a gene can correspond to multiple regions, in practice our enrichment tests used a set of terms with a similar or greater level of specificity. More broadly, though, the Zarin *et al.* clusters generally have stronger enrichments both in terms of their corrected *p*-values and the absolute number regions associated with a term (compare Table 1 in Zarin *et al.* to Table 1 in this manuscript). Though we cannot completely rule out other factors, *e.g.*, possibly a less robustly annotated *Drosophila* genome, we feel comfortable in our conclusion that the relationship between conserved features and function in IDRs is less pronounced in *Drosophila*.

It is true the log ratios of the likelihoods are independent from the values of the inferred optima. For example, this analysis can report a signal of conservation for fraction_Q in IDRs with both high and low fractions of glutamine. Furthermore, the different values of this feature may have different associated functional properties, which would diminish the power of the GO term enrichment analysis. We have added a sentence to the Discussion to acknowledge this caveat more explicitly.

A direct comparison of the sequence divergence in the alignments used in the yeast and *Drosophila* analyses is complicated by our choice to normalize the sum of branch lengths to account for regions with different numbers of sequences (compare Fig 1 supplement 1 in Zarin *et al.* to Fig S1 in this manuscript). Fortunately, the hypothesis testing analysis (Fig 4) and the observed type I error rates exceeding their nominal values (Fig 5) indicate there is sufficient divergence to detect signals of conservation. Additionally, we have expanded our description of the alignments and included a reference to the original work in the main text, which contains an extensive discussion of the selection of the *Drosophila* species.

We have created a clustering plot analogous to Fig 7 except using the means estimated from the OU model rather than the log likelihood ratios (Fig S15). Though there are some similar clusters in the two plots, particularly in the columns corresponding to the motif features, they are generally more fragmented or diffuse in the OU means plot.

COMMENTS FROM REVIEWER 2
Comment 1:
How much are the conclusions dependent on a correct prediction of disorder? The authors touches upon this question without addressing it:
"For each residue in the input sequence, AUCPreD returns a score between 0 and 1 where higher values indicate higher confidence in a prediction of intrinsic disorder. In some alignments, the scores vary by nearly this entire range at a given position even when there is a relatively high level of sequence identity"
Even with experimental data at hand the definition of an IDR may not be clear.

Response to Comment 1:
A similar concern was raised and addressed in Comment 2 from Reviewer 1.

Comments 2-5:
2. Title, write out IDR
3. There are a few grammatical errors/missing words here and there.
4. Beginning of Results, line 118; "Although both distributions span several orders of magnitude, the order regions are generally longer than the disorder regions, with means of 105 and 245 residues, respectively." Swap place of 105 and 245.
5. Line 185, isoelectric point

Response to Comments 2-5:
Thank you for these suggestions. We have incorporated Comments 2-4 in the text. We have chosen not to change “isopoint” to “isoelectric point,” as this line was specifically referring to the name of the feature corresponding to isoelectric point. Many features have abbreviated names for clarity, and we use these names through the text, *e.g.*, fraction_Q and repeat_K, so we believe changing our convention in this case may confuse readers.

COMMENTS FROM REVIEWER 3
Comment 1:
A few of additional papers/preprints the authors may wish to consider in the context of this work (generally) are the following, although none of these are required, they are

simply additional literature that I think support the authors conclusions and represent prior relevant work!

Response to Comment 1:
Thank you for these paper suggestions. We have reviewed them and added citations in the Introduction and Discussion where appropriate.

Comment 2:
Line 109
"We identified regions with high levels of inferred intrinsic disorder in over 8,500 high quality alignments of single copy orthologs from 33 species in the Drosophila genus using the disorder predictor AUCPreD".

If these alignments were pre-existing, it would be useful to briefly expand on where they came from and how they were generated. If they were created for this study, it would similarly be useful to expand on how they were generated. The fact these are single-copy orthologs is important, and it would be good for the authors to briefly expand on why this is the case and how this was ensured.

Response to Comment 2:
We have expanded this section with text explaining the origin of the alignments and a corresponding reference. We have also included a sentence that touches on the importance of using full-length single copy orthologs for these analyses.

Comment 3:
Line 119
"We then quantified the sequence divergence in each region by fitting phylogenetic trees to the alignments using amino acid and indel substitution models."

It would be useful to add one more sentence (and a reference or two?) explaining what this does and how it works (for a less expert audience).

Response to Comment 3:
We have moved a sentence that explains substitution models from the following paragraph to this section, so it appears alongside their first mention. We have also included a reference to a review of phylogenetic analysis and substitution models.

Comment 4:
Line 150
To what extent are the exchangeability matrices in Fig. 1 really the same, and differences are solely determined by differences in composition for the disordered/ordered regions? It seems - by eye, anyway - that, as stated in the text, the patterns are very similar, and while the log10 ratios show differences the major differences appear to be associated with the rarest amino acids, which I tend to think maybe noise more than a bona fide signal? For example, do we really think C->E/D is meaningfully different or is the log10 ratio of two very small numbers inherently sensitive to small fluctuations because the absolute values

are small? To be clear, this is not a problem, but I guess I'm unconvinced there is strong evidence for meaningful differences in exchangeability coefficients for residues in disordered vs. ordered regions (which is actually really interesting!).

Response to Comment 4:
In the substitution models used here, the exchangeability coefficients are distinct parameters from the background distribution of amino acids, so, in principle, they are independent. In practice, though, the rarity of certain amino acids or transitions between them can introduce significant variability in the parameter estimates as noted by Reviewer 3. This is true for some exchangeabilities we estimate where, as shown in Fig S6 panel B, their coefficients of variation are approximately 1.5, *e.g.* W <-> H. However, most exchangeabilities are estimated with much less variation, so we are confident the overall trends in the disorder-depleted and -enriched classes of residues we note in the text are meaningful.

Comment 5:
Line 180
I 100% agree with the authors stated concern re: comparing evolutionarily related sequences, but the description of what the 'contrasts' are here left me unsure of what exactly is going on. I'd appreciate it if the authors could expand a bit on this, perhaps even with a schematized figure explaining conceptually how this works (specifically expanding on the "general assumptions of the underlying evolutionary process, are independent and identically-distributed" [everything up until here is very clear]). It would also be helpful to consistently refer to 'disorder scores' as 'disorder scores' instead of alternating between 'disorder scores' and just 'scores'.

Response to Comment 5:
These are excellent suggestions. We agree the contrasts algorithm is often opaque to audiences who are not familiar with its technical implementation. Accordingly, we have created a new supplementary figure which illustrates the algorithm on a toy data set with three tips. We have also revised the text to use the phrase "disorder scores" consistently.

Comments 6-7:
Line 206
I think it would be extremely instructive to provide a short additional paragraph explaining algorithmically how the BM model is implemented. For example, is this done by taking a region, and then making small random sequence perturbations at the level of single amino acid substitutions at a rate defined by σ^2_{OU} where the substitutions are accepted/rejected based on the resulting deviation from μ_{bm}? Regardless, it would be useful to in just a few sentences explain the implementation here. Moreover, it wasn't clear to me if this process is done focussing on a single feature (i.e. μ_{bm} for a single sequence property) or if this is some kind of random walk through a high dimensional space. Again, I think this would be clarified by a few sentences just outlining the mechanics of how the BM model is implemented.

Line 245

Same comment as above here for the OU model, written ideally in a way that provides a clear comparison with the BM model.

Response to Comments 6-7:
Thank you for these suggestions. We note that the text already includes a detailed explanation of the implementation of both models in the Methods. We chose to keep these details, which are highly technical, in the Methods, as we felt they would distract from the key point that these are models with two or three parameters that govern the starting position of the random walk, the rate at which variation accumulates, and, for the OU model, the strength of selection. We agree the text can more explicitly state that the models are fit to each feature individually, and we have slightly re-worded three sentences in the Results and Methods to emphasize this point.

Comments 8-10:
For Fig S1. it might be useful to place y-axis on a log scale to make the distributions more visually accessible (this is just a suggestion, and the authors may disagree, which is fine!).

Fig 1.
Please label the matrices so we can understand what is being shown without needing to cross-reference against the figure caption.

Fig 3.D
Relating the many overlapping lines to the many similar colors here is very tricky. I suggest breaking the legend down and just moving each of the sequence feature terms to sit alongside the associated arrow so we, the reader, can do zero work and easily interpret which arrow reports on which feature.

Response to Comments 8-10:
Thank you for these suggestions. We respond to each individually below.

We agree that showing linear and log scales of histograms with long tails are helpful visualizations of the data, and we often include both when the overall layout of the figure permits. Based on Reviewer 3's suggestion, we tested a log scale in panel A of Fig S1, but we ultimately found the linear scale more useful for interpreting the relative numbers of regions in the interval excluding the tails.

We have added titles to the matrices as requested.

We agree some modifications to the panel can reduce the effort required to interpret it. However, we feel that labeling each of the arrows directly would crowd the plot and further confuse the reader. We instead have chosen to group the features into glutamine, charge, and glycine categories in the legend and only to label these categories on the plot itself. As these are the categories described in the text, we feel this choice will also improve the coherence between the text and the figure.

Sincerely,

Marc Singleton, PhD
Postdoctoral Fellow
Eisen Lab
University of California, Berkeley
Howard Hughes Medical Institute
